# Selective Targeting of Brain Tumors with Gold Nanoparticle-Induced Radiosensitization

**DOI:** 10.1371/journal.pone.0062425

**Published:** 2013-04-30

**Authors:** Daniel Y. Joh, Lova Sun, Melissa Stangl, Ajlan Al Zaki, Surya Murty, Phillip P. Santoiemma, James J. Davis, Brian C. Baumann, Michelle Alonso-Basanta, Dongha Bhang, Gary D. Kao, Andrew Tsourkas, Jay F. Dorsey

**Affiliations:** 1 Department of Radiation Oncology, Perelman School of Medicine, University of Pennsylvania, Philadelphia, Pennsylvania, United States of America; 2 Department of Bioengineering, School of Engineering and Applied Sciences, University of Pennsylvania, Philadelphia, Pennsylvania, United States of America; 3 Department of Cancer Biology, Perelman School of Medicine, University of Pennsylvania, Philadelphia, Pennsylvania, United States of America; University of Michigan School of Medicine, United States of America

## Abstract

Successful treatment of brain tumors such as glioblastoma multiforme (GBM) is limited in large part by the cumulative dose of Radiation Therapy (RT) that can be safely given and the blood-brain barrier (BBB), which limits the delivery of systemic anticancer agents into tumor tissue. Consequently, the overall prognosis remains grim. Herein, we report our pilot studies in cell culture experiments and in an animal model of GBM in which RT is complemented by PEGylated-gold nanoparticles (GNPs). GNPs significantly increased cellular DNA damage inflicted by ionizing radiation in human GBM-derived cell lines and resulted in reduced clonogenic survival (with dose-enhancement ratio of ∼1.3). Intriguingly, combined GNP and RT also resulted in markedly increased DNA damage to brain blood vessels. Follow-up *in vitro* experiments confirmed that the combination of GNP and RT resulted in considerably increased DNA damage in brain-derived endothelial cells. Finally, the combination of GNP and RT increased survival of mice with orthotopic GBM tumors. Prior treatment of mice with brain tumors resulted in increased extravasation and in-tumor deposition of GNP, suggesting that RT-induced BBB disruption can be leveraged to improve the tumor-tissue targeting of GNP and thus further optimize the radiosensitization of brain tumors by GNP. These exciting results together suggest that GNP may be usefully integrated into the RT treatment of brain tumors, with potential benefits resulting from increased tumor cell radiosensitization to preferential targeting of tumor-associated vasculature.

## Introduction

Glioblastoma multiforme (GBM) is the most prevalent and aggressive primary brain malignancy and carries a dismal prognosis. Multimodal therapies involving surgical resection, chemotherapy, and radiation therapy (RT) are currently considered the standard treatment for GBM, yet the median survival remains just over a year [1], [2]. A major hurdle in the clinical management of GBM is the blood-brain barrier (BBB), comprised of the specialized tight junctions and endothelia that line the central nervous system vasculature. The BBB restricts entry of many blood plasma constituents, including a host of circulating therapeutics.

Brain tumors themselves can disrupt BBB integrity to an extent, through mechanisms such as secretion of soluble factors that actively degrade tight junctions [3], as well as formation of abnormal blood vessels with defective expression of tight junction proteins such as occludin [4] and claudin [5]. Large and advanced brain tumors exhibit especially disrupted BBB integrity. This is due to loss of occludin [4] and extensive abnormal angiogenesis, which induces structural and functional alterations including increased endothelial permeability [6]. Indeed, tumor blood vessel walls often exhibit loss of integrity due to endothelial cell irregularity [7]; while this disruption may enable metastasis via tumor cell migration into vasculature, it may also allow for increased extravasation of blood-borne agents into tumor tissue. Such behavior is known as the enhanced permeability and retention (EPR) effect, attributed to the abnormal anatomy and physiology of tumors (i.e leaky vasculature, endothelial fenestrations, poor lymphatic drainage) [8]. However, previous studies have shown that peripheral areas of less advanced brain tumors often contain subregions with intact and especially robust BBB (or blood-tumor barrier, BTB), leading to cancer cell treatment resistance [9], [10]. Patchy variations in BBB permeability throughout a tumor can result in inconsistent and unpredictable dissemination of circulating drugs or radiosensitizers [11].

While targeted RT is already a mainstay of GBM therapy regimens, due to its ability to cause cancer cell death by inducing double-stranded DNA breaks, it also provides an intriguing strategy for modulating the permeability of the BTB and facilitating the delivery of therapeutic agents across it. Recent MRI studies have shown that radiation can increase the permeability of the BTB to gadolinium diethylenetriaminepentaacetic acid (MW∼470 kDa) in human patients [10]. Additionally, we have used RT-induced BBB disruption to enhance the delivery of drug-loaded nanopolymers (diameter ≈ 40–70 nm) to orthotopic animal models of human GBM [12]. These and other results suggest that targeted RT can enhance the uptake of circulating therapeutics in brain tumors by increasing the permeability of the BTB. In particular, although sufficiently large tumors can themselves disrupt the BBB, further RT-induced permeabilization of the BBB could allow accumulation of drugs in smaller and less disruptive tumors. The permeability-modulating effects of RT on the BBB, if coordinated with the efficient delivery of anti-cancer drugs to tumor tissue, would represent yet another therapeutic advantage of targeted radiation.

Nanometer-scale particles represent one such class of agents which could better extravasate into brain tumor tissue given a more permeable BBB. In particular, gold nanoparticles (GNPs) have recently received much attention as a potential tool in cancer treatment and diagnosis due to their low toxicity [13], [14], enhanced CT contrast capability[15]–[19], possibility of functionalization with various chemotherapies [20] or targeting ligands [21], and ability to enhance the efficacy of RT *in vitro* and *in vivo* [22]–[26]. GNPs intrinsically possess several characteristics that make them a promising candidate for permeability across the BBB, including small size, lack of tissue reactivity, and absence of charge [27], [28]. In particular, GNPs decorated with polyethylene glycol (PEG) have been shown to be highly uniform in diameter, stable under physiological conditions, and biocompatible [22], [29]. Importantly, these PEG-coated GNPs possess antibiofouling properties and thus exhibit prolonged systemic circulation half-life [16]. This can further promote EPR-driven uptake in tumors, especially those potentiated by RT-induced permeabilization of the BTB.

By using cellular and animal models of human GBM, we characterize several versatile ways in which GNPs can be incorporated as adjuvants into brain tumor radiotherapy paradigms. We first investigated the ability for GNPs to act as radiosensitizers and vascular dose-painting agents that can enhance DNA damage to both GBM cells and vascular endothelial cells. Next, we assessed whether this GNP radiosensitization translated into improved survival in mice with orthotopic GBM. Finally, we utilized RT-induced disruption of the BBB in intracranial tumors as a noninvasive strategy to enhance the passive accumulation of GNPs across the BBB into orthotopic GBM xenografts.

## Methods

### Gold Nanoparticle (GNP) Synthesis

For our experiments, we used GNPs that were surface-modified with polyethylene glycol (MW∼5000, Laysan Bio, Inc.). The GNPs were synthesized by the reduction of gold chloride with citrate, using the Turkevich method [30]. Prior to synthesis, all glassware and stir bars were cleaned using aqua regia then rinsed thoroughly with Millipore water. Briefly, aqueous sodium citrate (15 mL, 55 mM) was added to a boiling solution of 60 mg of HAuCl_4_ (Sigma-Aldrich) dissolved in Millipore water (200 mL). The GNP solution was filtered using a 0.2 µm pore size nylon filter system (Millipore), and methoxy-terminated PEG thiol (MW∼5000, Laysan Bio Inc.) was added at a 4∶1 mass ratio (PEG:HAuCl_4_) and stirred overnight. The GNP solution was then purified from excess reactants, by washing them 7 times with 1X phosphate buffered saline (PBS), using 50 K MWCO Amicon centrifugal filter devices (Millipore).

### GNP Characterization

GNP stock samples were diluted in Millipore water and deposited on 200 mesh carbon coated copper grids (Polysciences, Warrington, PA) for TEM imaging using a JEOL 1010 transmission electron microscope operating at 80 kV. Stock samples of GNPs were diluted in pH 7.4 1X PBS for measuring the hydrodynamic diameter of the nanoparticles by dynamic light scattering (DLS). These measurements were acquired using a Zetasizer Nano-ZS (Malvern Instruments, Worcestershire, UK) using the non-invasive back-scatter (NIBS) mode. Zeta potential measurements were carried out by diluting GNP stock samples in in pH 7.4 1X PBS and the mean particle zeta potential was measured using a Zetasizer Nano-ZS. Concentration was determined by using both inductively couple plasma mass spectrometry (ICP-MS) and a Cary Bio 100 UV visible spectrophotometer (Varian, Agilent).

### Cell Culture

The cellular and animal studies described here utilized the human U251 glioblastoma cell line (ATCC), which has been shown to emulate, in orthotopic murine models, the relevant pathobiological features of GBM encountered in humans [31]. These U251 cells were genetically engineered to express firefly luciferase, enabling serial bioluminescence imaging measurements of GBM proliferation *in vitro* and *in vivo* [32], [33]. Cells were cultured in Dulbecco’s Modified Eagle Medium (DMEM) supplemented with 10% fetal bovine serum and 1% antibiotics (Invitrogen), and kept in a tissue culture incubator at 37°C and 5% CO_2_.

### Orthotopic Xenografts and Tumor Bioluminescence Imaging

Mouse work was performed under a protocol approved by the Institutional Animal Care and Use Committee (IACUC) at the University of Pennsylvania (protocol #804318). A stereotactic xenograft implantation and monitoring procedure previously described [32], [33] was used to implant mice (nude female athymic mice (NCI Production)) with GBM tumor cells. Briefly, after mice were anesthetized by intraperitoneal injection of a ketamine/xylazine mixture (at a dose of 140 mg/kg and 10 mg/kg respectively), they were immobilized on a Stoelting Digital Just for Mouse Stereotactic platform (Stoelting Inc.) via a bite bar, nose clamp, and ear bars. A 0.45 mm burr drill was used to drill a hole into the skull 2 mm posterior and 1.5 lateral to the bregma, visualized after cutting the overlying skin. 7 µl of U251 cells (50,000 cells/µl) were injected into the burr hole at a depth of 3 mm, at a rate of 0.5 µl/minute, with careful drying of the skull using a microsurgical sponge spear to remove any tumor-containing fluid that might reflux out of the burr hole during implantation. Finally, bone wax and veterinary tissue glue were applied to seal the wound.

After stereotactic implantation, tumor growth was monitored every other day with bioluminescent imaging to assess size and ensure that the tumor remained intracranial with no metastasis. After induction of anesthesia with 2% isoflurane and oxygen, mice were intraperitoneally injected with 100 µL of D-luciferin potassium salt diluted in PBS to a concentration of 50 mg/mL. They were then placed into a bioluminescent imaging scanner (IVIS Lumina II, Xenogen Inc.) with constant flow of isoflurane through a nose cone to ensure maintenance of anesthesia. The luciferase-expressing U251 cells react with the injected luciferin, appearing as luminescent signal on photographs taken by the Living Image software. Tumor size was monitored by measuring bioluminescence intensity (photons/sec/steradian/cm^2^). Prior work in our laboratory and by others has shown that the measured luminescence increases proportionally with the number of GBM cells present, and provides a reliable indication of tumor progression [32], [33]. Mice were monitored daily to ensure clean cages, adequate food and water, and good body condition. Mice were sacrificed if they exhibited excessive weight loss (>20%), tumor metastasis, lethargy, or other signs of distress consistent with IACUC standards.

### RT Administration

To deliver radiation therapy to both *in vitro* and *in vivo* models, we used the Small Animal Radiation Research Platform (SARRP), which is capable of delivering a CT image and a stereotactically guided dose of radiation by use of collimators. For *in vitro* radiosensitization experiments, GBM cells in chamberslides received 4 Gy (150 kVp), administered through a wide circular field 11 cm collimator. For *in vivo* experiments, mice undergoing RT received 20 Gy (175 kVp) to the brain, stereotactically administered through a 1.5 cm collimator.

### *In vitro* Assays of GNP Radiosensitization

Prior to irradiation, GBM cells in chamberslides were incubated with GNP (1 mM suspension in DMEM) for 24 hours, and another group of cells was treated with vehicle. GNP and vehicle-treated cells were then mock-irradiated or irradiated with 4 Gy RT with the SARRP (150 kVp). To quantify RT-induced DNA damage to cells, we immunofluorescently labeled cells for gamma-H2AX (γh2ax). This biomarker for double-strand DNA breaks (DSBs) allows for the visualization of discrete foci, whose density in the nucleus is proportional to the amount of unrepaired DNA DSBs in the cell [32]. Subsequent imaging to assay DNA damage was performed with deconvolution microscopy (Applied Precision, Inc.) using a 60x (1.42 NA) objective lens. Quantification of γh2ax foci density was performed with a custom macro in ImageJ, in which foci were counted after applying a top-hat filter and constant value threshold based on unirradiated controls, similar to the approach described by Hou et al. [34].

For clonogenic survival assays, cells were plated at predetermined densities in 60 mm dishes and treated with vehicle or 1 mM GNPs for 24 hours, then mock-irradiated or irradiated with 4 Gy RT. Cells were then kept in a humidified incubator for 10–14 days. Subsequently, they were stained with crystal violet and the resulting colonies counted. A colony was defined to contain greater than 50 cells. The surviving fraction (SF) was calculated as SF = (# surviving colonies)/(# cells plated×plating efficiency). Curves were fitted to a linear quadratic (LQ) equation, in which the SF was approximated by SF = exp[–αD–βD^2^], and the sensitizer enhancement ratio (SER) was calculated as the ratio of the SF curve with GNPs over that without.

### Vascular Dose Painting with GNPs

To assess the vascular dose painting (VDP) effect, RT was delivered to the mouse brain at peak circulating GNP concentrations, within 5 minutes of intravenous injection. 48 hours after irradiation (20 Gy, 175 kVp), mice were given an overdose of 140 and 10 mg/kg ketamine and xylazine, respectively, and transcardially perfused with 1X TBS (Quality Biological, pH7.4, 10X) with zinc fixative (BD Pharmigen). The brains were removed and post-fixed for 3–4 days in fixative and then sucrose. Brains were frozen with isopentane in O.C.T Compound (Tissue-Tek Sakum-Fineteck) at −30–40°C and then sectioned at 10 µm slices. Slide sections were rinsed with PBS and cells were permeabilized with 0.5% Triton-X 100 (Sigma-Aldrich) in 1X PBS for 2 minutes. After rinsing with PBS, each tissue section was given 100 µL of streptavidin solution (Vector Laboratories) and incubated in a moist chamber at room temperature for 15 minutes. After rinsing with PBS, each tissue section was given 100 µL Biotin solution and incubated in a moist chamber at room temperature for 15 minutes. After another PBS rinse, tissue sections were incubated in 5% goat serum (Vector Laboratories) in 1X PBS in a moist chamber at RT for 30 minutes and then the buffer was decanted. CD31 primary antibody (rat anti-mouse CD31, BD Pharmingen) at 1∶100 dilution in 1X PBS and 1% Bovine Serum Albumin (Sigma) and biotinylated γh2ax (Millipore) at 1∶500 dilution in 1X PBS and 1% bovine serum albumin (BSA) was added to each tissue section, and the tissue sections were incubated overnight at 4°C.

After rinsing with PBS in a dark room, cells were incubated with goat anti-rat Alexa 488 secondary antibody at 1∶200 dilution in 1X PBS and 1% BSA and Streptavidin Conjugate (Molecular Probes) at 1 µg/ml in 1X PBS and 1% BSA for 1 hour at room temperature. Following a final rinse in PBS, the slides were mounted with anti-fade medium containing DAPI and coverslips. Fluorescent imaging of γh2ax foci and CD31 distribution was performed using widefield (10x, 0.35 NA) epifluorescence microscopy (Nikon TE2000). The colocalization between γh2ax and CD31 images were analyzed by calculating Mander’s coefficient using ImageJ. In brief, CD31 and γh2ax-positive cells were identified using intensity thresholding (determined from unirradiated control images) and then converted to binary images. The CD31 and γh2ax colocalization was then quantified using Mander’s coefficient (M2) provided in the ImageJ colocalization analysis package, which calculates M2 as the summation of all colocalized γh2ax/CD31-positive pixels divided by the sum of all CD31-positive pixels.

### GNP Radiosensitization of Brain Endothelial Cells

Endothelial cells (ECs) were isolated from mouse brain by immunoseparation beads. Filtered cells from the brain were incubated with microbeads conjugated to an anti-biotin, anti-CD31 antibody specific for ECs, and then run through a magnetic collimator to separate non-endothelial from endothelial cells. To mimic the GBM microenvironment, ECs were co-cultured together with U251 cells in advanced DMEM and HEPES (Invitrogen), using a 1∶7 EC:U251 cell ratio, and radiosensitization experiments were performed with GBM cells as described above. Duplicate wells of co-cultured endothelial and U251 cells were treated with GNP alone, RT alone, and GNP followed by RT. DNA damage was assayed by immunofluorescent staining for γh2ax using the same streptavidin-biotin antibody described above, followed by deconvolution imaging to visualize foci.

### Survival Analysis of Xenograft-implanted Mice Treated with RT and GNP

GBM tumors were stereotactically implanted into mice and allowed to grow to a radiance of 1×10^8^ p/sec/cm^2^/sr as measured by BLI. Next, brain tumor-bearing mice were split into two treatment groups of 5 each–one group received 20 Gy RT only; and the other received IV GNP 48 hours before a single dose of 20 Gy RT. Mice were monitored for tumor growth and weight, and were sacrificed upon loss of 20% body weight. Survival time to this endpoint was calculated from date of treatment. Survival data were plotted using Kaplan-Meier techniques.

### Inductively Coupled Plasma-Mass Spectrometry (ICP-MS) Analyses

Forty-eight hours after intravenously injecting 100 µL of concentrated GNPs into mice with normal or tumor-bearing brains, right cerebral hemispheres were extracted (RH; ipsilateral to the tumor inoculation site) and analyzed for gold content with inductively coupled mass spectrometry (ICP-MS), which reports the mass of gold per gram of tissue. Next, the effect of RT on this uptake was assessed by administering 20 Gy RT between 7 to14 days prior to the IV GNP injection and ICP-MS analysis in the same manner as above (*n* = 4 for tumor-bearing brains or *n* = 3 for healthy brains for each ICPMS study). Also, in brain tumor-bearing mice treated with RT in addition to GNP, the tumor itself was carefully extracted from the brain parenchyma and subjected to ICP-MS analysis, in order to assess true rate of GNP accumulation into the tumor only.

## Results

### GNP Synthesis and Characterization

**Figure 1:**
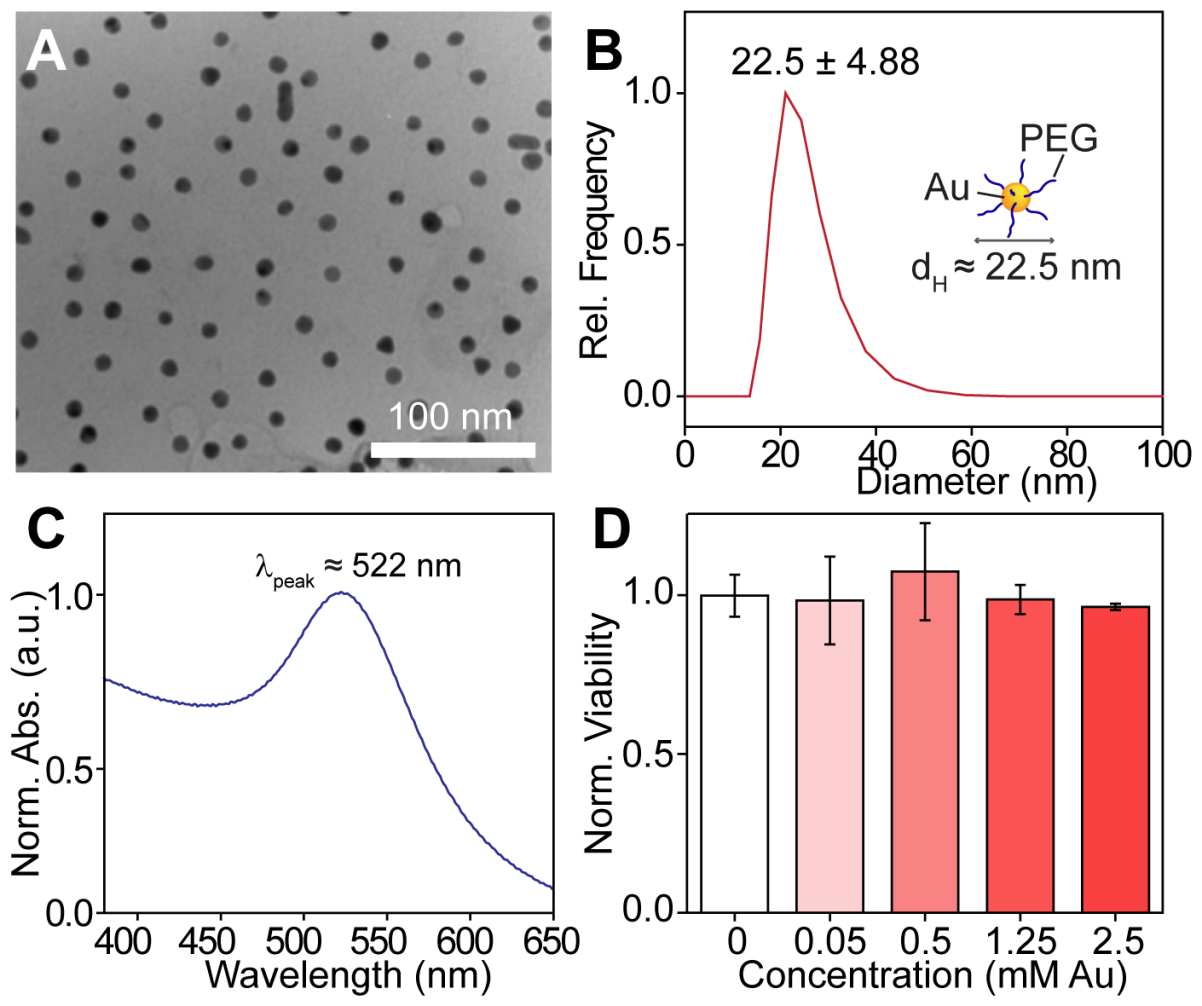
Gold nanoparticle characterization. **A.** Transmission electron micrograph of GNPs having approximately 12 nm cores. **B.** Representative dynamic light scattering measurement of GNPs. Data was fit to a Gaussian function to determine the peak ± s.d. of nanoparticle hydrodynamic diameter (d_H_). **C.** UV-vis absorption spectrum of GNPs showing characteristic surface plasmon resonance at λ ≈ 522 nm. **D.** MTT viability assay of U251 cells treated with increasing concentrations of GNPs for 24 hours. Error bars, mean viability ± s.d. of three replicates.

Our synthesis approach yielded highly monodisperse GNPs with approximately 12 nm gold cores and hydrodynamic diameters (d_H_) of approximately 23 nm, as evidenced by transmission electron microscopy (Figure 1A) and dynamic light scattering measurements (Figure 1B). UV-vis spectroscopy showed that the nanoparticles exhibited strong absorption peaks at ∼522 nm resulting from their characteristic surface plasmon resonance (inset, Figure 1C). Incubating U251 cells with increasing concentrations of these surface-modified GNPs for 24 hours in the absence of radiation did not significantly affect their viability (up to 2.5 mM Au) as measured through MTT assay (Figure 1D).

### GNPs Radiosensitize Human GBM Cells

**Figure 2:**
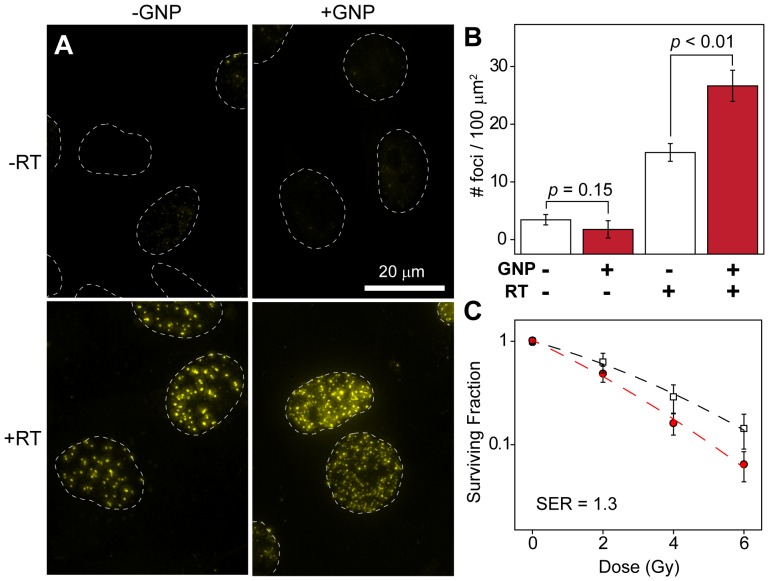
Assessing GNP enhancement with *in vitro* assays of radiosensitivity. **A.** Deconvolution imaging of γh2ax foci in U251 cells that were mock-irradiated (upper) or irradiated with 4 Gy (lower). Cells irradiated with 1 mM GNPs display a 1.7-fold higher density of persistent γh2ax foci 24 hours after RT. **B.** Quantitative analysis of γh2ax foci for N >100 viable nuclei. Error bars, 95% confidence interval. Statistical significance was determined using a two-tailed *t*-test (α = 0.05), with *p*<0.05 being considered significant. **C.** Clonogenic assay of U251 cells treated with (red circles) and without (hollow squares) 1 mM GNPs and given radiation doses of 0, 2, 4 and 6 Gy. Error bars represent the mean survival ± s.d. of at least four replicates.

Compared to untreated controls, GNP administration alone did not significantly increase the γh2ax expression in U251 cells in the absence of radiation (*p = *0.15) (upper panels, Figure 2A). This indicates that GNPs alone do not significantly induce DNA damage. However, GNP-incubated cells treated with RT displayed an increased density of γh2ax foci compared to cells receiving irradiation alone (lower panels, Figure 2A) (*p*<0.01). Calculation of γh2ax density for cells in each treatment group suggest that RT with GNPs led to 1.7-fold increase in γh2ax density in U251 cells compared those treated with RT only (Figure 2B), suggesting that gold can significantly enhance radiation cytotoxicity in human GBM cells. Furthermore, clonogenic survival assays showed decreased survival in GBM cells irradiated with GNPs in a dose-dependent manner compared to those receiving irradiation alone (Figure 2C). Using the linear-quadratic model to assess the enhancement of radiation effects, we estimated that GNPs produced a sensitizer enhancement ratio (SER) of approximately 1.3 for U251 human GBM cells *in vitro*.

### GNPs Induce Endothelial Cell Radiosensitization and Vascular Dose Painting (VDP)

**Figure 3:**
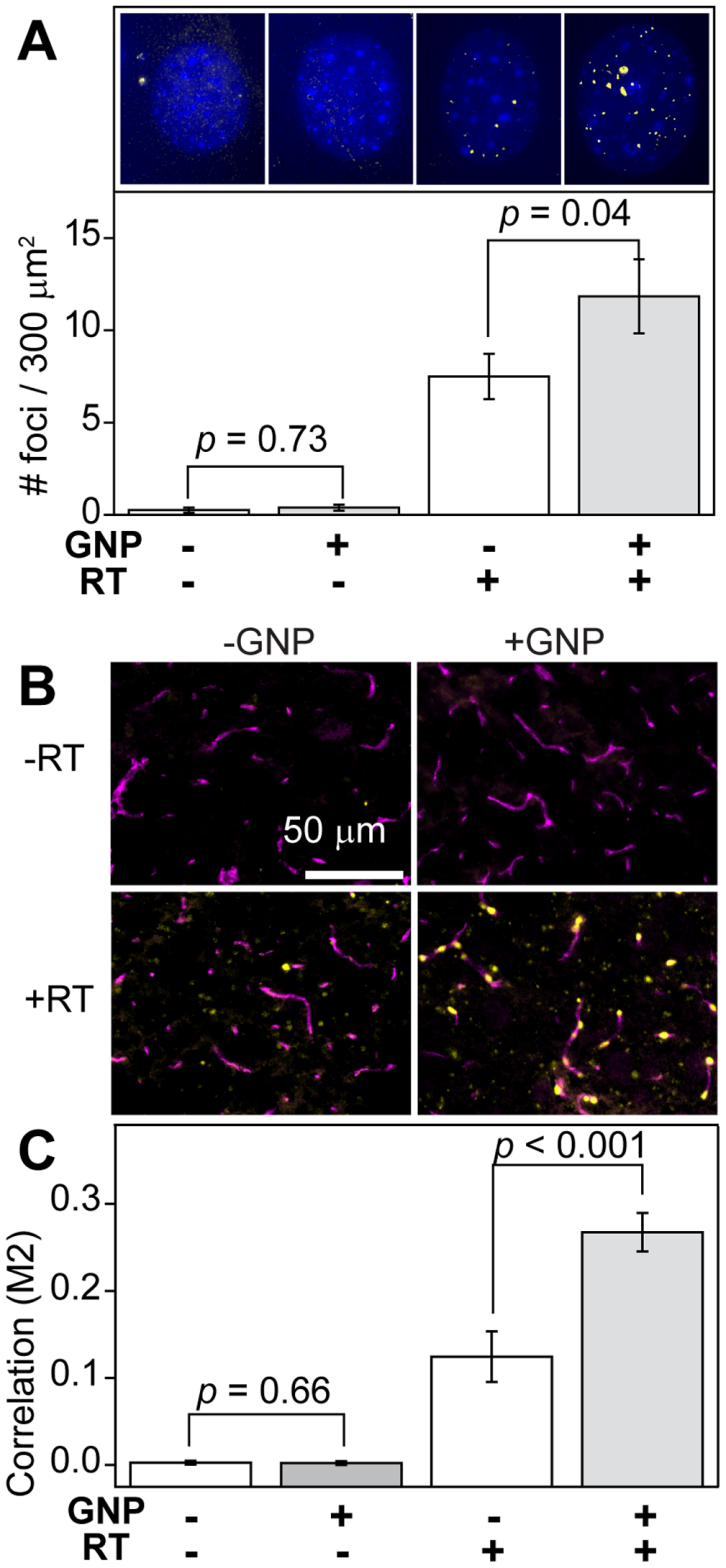
Visualization of vascular dose painting effects via immunofluorescent labeling of DNA DSBs and vascular endothelial markers. **A.** Mouse brain endothelial cells co-cultured with human GBM cells (1∶7, respectively) *in vitro* show enhanced RT damage when irradiated (4 Gy) with 1 mM GNPs. Upper : Immunofluorescence imaging of γh2ax foci and DAPI in normal mouse brain endothelial cells with the indicated treatments. Lower: Quantitative analysis of γh2ax foci (yellow) for N >10 viable nuclei (blue) of normal murine brain endothelial cells co-cultured with human GBM cells *in vitro*. Error bars, 95% confidence interval. Statistical significance was determined using a one-tailed *t*-test (α = 0.05), with *p*<0.05 considered significant. **B.** Brains irradiated immediately following GNP injection leads to considerable colocalization of DNA DSB and blood vessels compared to those receiving RT alone. Healthy brains were mock-irradiated or irradiated with 20 Gy (whole-brain) immediately (<5 min) after i.v. administration of 1.25 g Au/kg GNPs or saline. Mice were sacrificed 24 hours later, and their brains were fixed/stained for γh2ax, CD31, and DAPI. C. γh2ax colocalization with CD31-positive cells was performed by calculating Mander’s coefficient (M2) in binary projections of CD31 and γh2ax channels.

We next performed a series of experiments on brain-derived endothelial cells to determine if GNPs could produce a similar radiosensitizing effect to that seen in GBM cells (Figure 3). When co-cultured with GBM cells (1∶7, respectively), brain endothelial cells showed a 1.5-fold increase in γh2ax density after treatment with GNP followed by RT (*p* = 0.04) compared to those irradiated without GNPs (Figure 3A). No significant difference in γh2ax foci was seen in unirradiated cells that were treated with or without GNPs. We next sought to confirm the radiosensitzing effect of GNPs on endothelial cells *in vivo* and begin to characterize the potential VDP characteristics of GNPs in combination with radiation. The bottom panels in Figure 3B are representative sections of brain stained in the recovery phase (24 hours) following whole-brain irradiation (20 Gy), treated (left) and untreated (right) with GNP injection immediately prior to RT. The upper panels represent unirradiated controls that were likewise GNP-treated or untreated. As expected, these unirradiated controls showed positive staining for CD31 but no appreciable staining for γh2ax, which confirms that GNPs alone do not induce DSBs. The bottom left panel, brain irradiated in the absence of gold, demonstrates γh2ax foci, but minimal colocalization of γh2ax-positive cells with vascular structures (CD31 staining), indicating that the RT-induced DNA damage is diffusely distributed across the brain parenchyma. However, as shown in the bottom right panel, irradiating the brain at peak circulating systemic gold concentrations leads to a strikingly high colocalization of regions that positively stain for both γh2ax and CD31, suggesting a preponderance of unrepaired DNA damage localized to endothelial cells. By quantifying γh2ax/CD31 colocalization (using Mander’s coefficient) (Figure 3C), these data suggest that the overlap of γh2ax and CD31-positive signals is significantly higher in samples receiving GNP and RT than samples receiving RT alone. Taken together, these *in vivo* and *in vitro* results suggest that GNPs may preferentially enhance RT-induced DNA DSB damage to brain endothelial cells.

### Orthotopic Mouse Models of Human GBM using Bioluminescent U251 Cells

**Figure 4:**
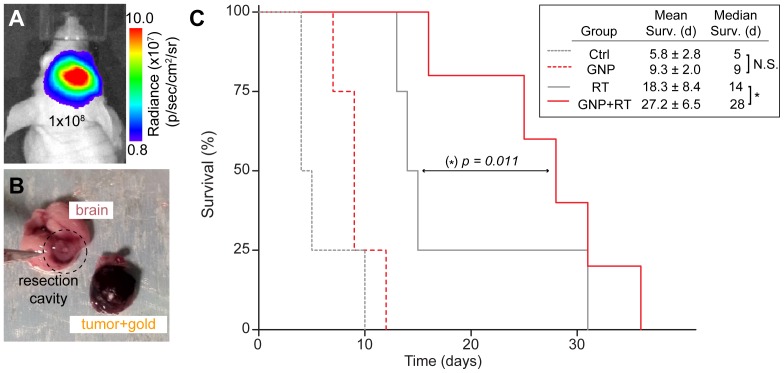
GNP administration in combination with RT improves survival in mice with advanced GBM tumors. **A.** BLI of a representative mouse with advanced orthotopic GBM xenografts (radiance ∼10^8^ p/sec/cm^2^/sr) used for the survival study. **B.** Photograph of a brain and resected tumor 48 hours after intravenous injection of GNPs. Tumor shows darkened appearance due to extravasation due to EPR into the tumor. **C.** Survival data in mice with advanced orthotopic GBM treated with or without GNPs followed by mock-irradiation or given stereotactic RT (20 Gy). The right cerebral hemispheres of nude mice were initially implanted with 350,000 U251 cells, and tumors were allowed to grow until the measured radiance reached ∼10^8 ^p/sec/cm^2^/sr (approximately 3–5 weeks post-implantation), at which point the mice were given their respective treatments (*n* = 5 for GNP+RT and *n* = 4 for control, GNP, and RT groups). Median and mean survival analysis were obtained with Kaplan-Meier analysis, and comparison between RT versus GNP+RT survival curves showed *p* = 0.011. Mean survival times are shown with 95% confidence intervals. N.S. in the figure indicates lack of statistical significance, while the asterisk (*) denotes that significance was reached (α = 0.05).

**Figure 5:**
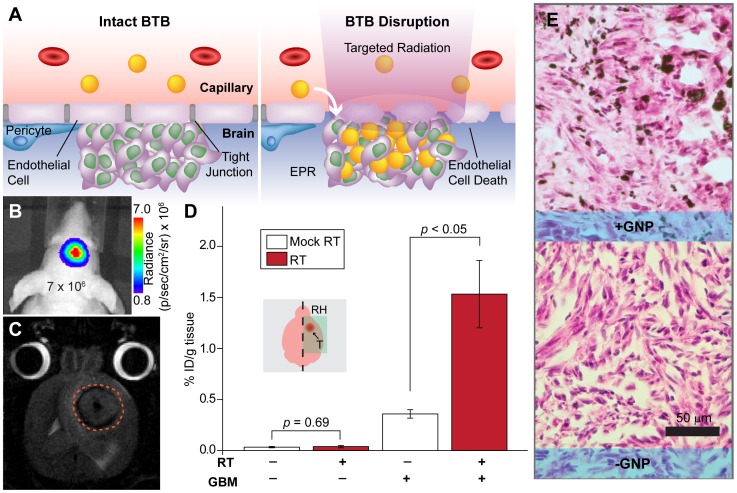
Radiation-induced modulation of the blood-brain barrier leads to increased uptake of GNPs in orthotopic GBM xenografts. **A.** Schematic of BBB-disruption with targeted RT, which leads to endothelial cell death and loosening of tight junctions. **B.** Representative BLI image of a smaller, less disruptive GBM tumor (max BLI ∼10^6^) used in this experiment. **C.** T2-weighted MRI image of a stereotactically implanted intracranial GBM tumor (approximated by dashed orange line). **D.** ICP-MS analysis of gold uptake in the right hemispheres of healthy brains and those with orthotopic GBM excised from mice 48 hours after i.v. injection of saline or 0.4 g Au/kg GNPs administered 7–14 days after 20 Gy RT or mock-irradiation. The right cerebral hemispheres of mice were orthotopically inoculated with 350,000 U251 cells. Tumors were allowed to grow until the measured BLI irradiance reached ∼10^6^ p/sec/cm^2^/sr (approximately 2 weeks post-implantation), at which point the mice were given their respective treatments. Brains with tumors receiving RT (*n* = 4) prior to GNP administration show significant increase in EPR-driven gold accumulation compared to mock-irradiated controls (*n* = 4). Mock-irradiated (*n* = 3) and irradiated (*n* = 3) healthy brains, however, show no significant difference in gold uptake, suggesting that normal tissue may recover more quickly than tumor. **E.** Representative H/E staining of sections from orthotopic tumors with (+) and without (−) GNP injection.

Representative BLI signals in mice bearing orthotopic U251 xenografts used in our experiments are shown in Figure 4A, a large tumor (10^8^ p/sec/cm^2^/sr), and Figure 5B, a less advanced tumor (10^6^ p/sec/cm^2^/sr). The light emission is highly localized to the tumor inoculation site, confirming the absence of tumor dissemination to other areas such as the spine. As reported previously [35], we verified that our tumor models closely recapitulated histological phenotypes consistent with those of human GBM; and we provide a representative T2-weighted MRI image of an implanted orthotopic GBM tumor in Figure 5C.

### Increased Survival of Mice with Advanced GBM Treated with RT plus GNPs

We observed that intravenously injected GNPs can readily extravasate into advanced brain tumors, an example of which is shown in Figure 4B. As depicted in the survival curve in Figure 4C (*n* = 5 for GNP+RT and *n* = 4 for control, GNP, and RT groups), mice with advanced tumors receiving RT exhibited prolonged survival compared to those receiving mock-RT. Furthermore, GNP followed by RT experienced prolonged median survival time (28 days on average) compared to mice receiving RT only 14 days (*p* = 0.011). In general, mice receiving dual-modality treatment also displayed more normal activity and less weight loss than untreated or single-treated mice. These results preliminarily suggest that GNP successfully extravasated into the brain due to tumor-induced disruption of the BBB, and subsequently radiosensitized tumor cells to RT, leading to increased tumor cell killing and increased survival.

### GNPs Accumulate in GBM Tumors with Increased Uptake after RT

We next sought to determine whether RT-induced disruption of the BBB could allow more efficient accumulation of GNPs in smaller and less disruptive tumors (Figure 5A). Figure 5D shows that brains with less advanced GBM tumors are more permeable to GNPs compared to normal brain, as seen in the modest increase in gold content measured by ICP-MS. Radiation increased this uptake more considerably–brain tumor-bearing mice receiving 20 Gy RT prior to gold injection (approximately 7 to 14 days) exhibited higher gold uptake in the right cerebral hemisphere compared to the unirradiated control mice. Indeed, ICP-MS performed on samples comprised exclusively of tumor from the mice receiving GNPs two weeks post RT showed that these xenografts accumulated on average 3.7±1.9% ID/g tissue of gold. As shown in Figure 5E, histological sections of brain tumors stained with H&E readily demonstrate GNPs extravasating through tumor vasculature and penetrating into the surrounding tumor stroma.

## Discussion

The tumor penetration and radiosensitizing properties of GNPs discussed thus far suggest that engineered nanoparticles may provide a potentially useful adjuvant to RT in GBM management. First, we demonstrate that GNPs can effectively radiosensitize GBM tumor cells to subsequent RT, leading to enhanced DNA damage (as shown *in vitro*) and delayed tumor growth and improved survival (as shown *in vivo*). Endothelial cell and vascular dose painting experiments also support the notion that GNPs could be used to enhance radiation damage to tumor-associated vasculature, which we speculate would precipitate vascular shutdown, leading to extensive tumor cell death. Finally, although large and advanced GBM brain tumors disrupted the BBB sufficiently to allow extravasation of GNPs, we provide evidence that RT could further permeabilize the BBB and enhance accumulation of GNPs in tumor tissue, especially in smaller and less disruptive tumors. Taken together, these experiments suggest that GNPs and RT may be effectively leveraged in combination treatment regimens in a complementary manner leading to increased treatment efficacy.

The possibility of utilizing GNPs in clinical practice as adjuvants to RT as described here and elsewhere requires further characterization of clinically relevant parameters, including those relating to toxicity, pharmacokinetics, biodistribution, and radiosensitizing potential of various GNP formulations. However, the future for these agents appears promising. Notably, gold has been used in medical practice throughout history and continues today as a treatment for certain conditions, such as rheumatoid arthritis [36]. Reassuringly, when 12.5 nm GNPs were administered intraperitoneally into mice every day for 8 days, no evidence of toxicity was observed in any of the serial parameters followed for a subsequent two-plus months, including survival, behavior, animal weight, organ morphology, blood biochemistry, and tissue histology [13]. One potential concern with the use of GNPs may be protracted elimination from the liver[37]–[39] – with one study reporting 9% decrease in the content of gold in the liver from day 1 to 6 months, following the intravenous injection of 40 nm GNPs [40]. However, this potential concern has not prevented the use of gold in patients with poor cancer prognoses. In fact, several GNP formulations have already entered clinical trials for cancer treatment, including CYT-6091 (27 nm citrate-coated GNPs bound with thiolated PEG and TNFalpha) [41], and AuroShell® particles (∼150 nm, silica core with a gold shell, clinicaltrials.gov identifier # NCT00848042).

Given that the pharmacokinetic behavior GNPs depends greatly on the morphology and surface coating [28], [42], [43], rigorous quality control measures must be demonstrated prior to clinical translation of GNP-assisted strategies to minimize sample variations that may affect the outcome of therapy [44]. For instance, both computation[45]–[47] and experiment [26], [48] have suggested that GNP morphology may influence the extent of radiation-induced free radical formation and degree of radiosensitization, and Cho et al. demonstrated that morphology can also significantly modify the tissue kinetics and distribution properties [42] as well. Such considerations are particularly important for targeted GNP formulations, in which successful coupling of targeting ligands to the nanoparticles greatly affects the extent to which the GNPs can extravasate into tumor tissue, especially those tumor cells that are difficult to access (such as hypoxic tumor cells located relatively far from blood vessels or those in privileged organs such as the brain). Notably, our approach utilizing stereotactic RT to disrupt the BBB may provide an intriguing therapeutic strategy to further modify biodistribution profiles of various GNP formulations and should be investigated in greater detail.

Timing of RT in relation to GNP administration may be of utmost clinical significance in several of our hypothesized therapeutic mechanisms. Previous theoretical studies suggest that higher circulating GNPs levels yields greater vasculature-targeted RT damage [49], [50]; thus, RT may need to be administered immediately following a GNP administration. In contrast, to directly target tumor cells, intravenously injected GNPs need more time to extravasate into tumor stroma via the EPR effect. In this study, we waited 2–4 days after nanoparticle injection to administer RT to mice in the survival study; however, more work is needed to optimize delivery times and dosages. Similarly, in delivery-enhancing experiments here and elsewhere that used RT to permeabilize the BBB [35], we waited 7–14 days after RT to inject GNPs, but time-course experiments are needed to find optimal BBB permeability following RT, which would presumably be the time window targeted for delivery of GNPs.

Several limitations of this study should be noted. Though the U251 cell line used in this study has been shown to recapitulate features of GBM tumors found in humans, the physiological differences in our murine model and stereotactic implantation technique makes it impossible to completely replicate the *in vivo* environment of a GBM tumor arising spontaneously in a human. In addition, the endothelial cells isolated and cultured together with U251 cells are likely not entirely representative of tumor vasculature *in vivo*, since it is very challenging to recapitulate the disordered tumor microenvironment *in vitro*. Though preliminary data shows minimal *in vitro* and *in vivo* toxicity of the GNP formulation used in this study, more studies are needed to confirm the safety and biocompatibility of potential therapeutic agents.

Although we have focused in this study on radiosensitizing and therapeutic applications of GNPs in combination with RT, the paradigm of EPR and RT-induced BBB permeabilization is also extremely relevant to diagnostic and imaging modalities, such as MRI and CT. Having established that GNPs can accumulate in tumor tissue from intravenous injection, different formulations of nanoparticles can serve as contrast agents, and enhance imaging and diagnosis of brain tumors [51]. Thus, GNPs multiplexed with highly sensitive imaging agents may have great potential as a “theranostic” agent with multimodal applications in diagnosis and treatment of GBM [19], [51], [52].

Enhancing the delivery of GNPs to brain tumor tissue would open up a wide array of exciting diagnostic and therapeutic possibilities. Diagnostically, gold’s high atomic number [18] and capability for conjugating with MRI-active agents [53] could enable sensitive and specific multi-modal imaging of tumor tissue and boundaries [51]. From a treatment standpoint, one possible strategy for translating the RT-assisted delivery of GNPs into brain tumors into therapeutic gain is to subsequently use them as radiosensitizers which persist in tumor tissue. GNPs that have extravasated into tumor stroma can sensitize cancer cells to RT by propagating free radical formation, which induce DNA damage within tumor cells [54]. Several studies have previously demonstrated the radiosensitizing effects of various formulations of GNPs in both cellular and animal studies[23]–[26], [54]–[56].

In addition to directly augmenting radiation-induced DNA damage to GBM cells, GNPs may complement RT by localizing radiation damage to tumor-associated endothelial cells. Tumor cells secrete VEGF and other growth factors in order to induce and protect vascular endothelial cells, which in turn supply tumor tissue with oxygen and nutrients [57]. Targeting RT to tumor-associated endothelial cells with GNPs can break this tumor-protective cycle–damaged endothelial cells and vasculature will fail to deliver nutrients to tumor tissue, leading to ischemic necrosis and reduced VEGF production. Ngwa et al have proposed that GNPs can serve as tumor “vascular dose-painting” (VDP) agents [49], [58]–GNPs actively circulating through the abnormal and tortuous tumor blood vessels can locally enhance the dose of radiation absorbed by endothelial cells lining the vascular walls. Such endothelial cell damage may also facilitate the loosening of tight junctions in the BBB with lower radiation doses. These strategies, which depend on the timing between GNP administration and RT, represent yet another potential strategy for using GNPs to complement radiotherapy.

Ultimately, RT and GNPs may be able to work as synergistic anti-tumor approaches–RT can increase BTB permeability for better delivery of GNP into tumor stroma; these GNPs can in turn radiosensitize tumor cells as well as tumor-associated endothelial cells to RT-induced DNA damage. Such a scenario may be particularly relevant to fractionated RT regimens, whereby nanoparticles can be given together with sequential administrations of RT, and the two agents in conjunction can mutually compound their individual therapeutic effects over the course of a treatment regimen. Our experiments demonstrate that together with RT, engineered GNPs and their enhanced penetration through the BBB may provide novel and versatile avenues for anti-GBM therapy.

### Conclusions

Nanoparticles as adjuvants to radiation therapy may increase the efficacy of anti-GBM therapies. Specific intra-tumoral accumulation, especially combined with radiation-induced BBB disruption, result in augmented delivery of nanometer-scale agents to brain tumors in a targeted manner. In addition, by leveraging the radiosensitizing qualities of GNPs with RT-assisted delivery across the BBB demonstrated in this work, we provide a novel strategy for enhancing the efficacy of nanoparticle agents against brain tumors such as GBM which are currently incurable with standard treatment modalities. Further studies and optimization are needed to move closer to clinical implementation.
